# Decrease in expression of bone morphogenetic proteins 4 and 5 in synovial tissue of patients with osteoarthritis and rheumatoid arthritis

**DOI:** 10.1186/ar1923

**Published:** 2006-03-15

**Authors:** Carsten P Bramlage, Thomas Häupl, Christian Kaps, Ute Ungethüm, Veit Krenn, Axel Pruss, Gerhard A Müller, Frank Strutz, Gerd-R Burmester

**Affiliations:** 1 Department of Medicine, Nephrology and Rheumatology, Georg-August-University Göttingen, Robert-Koch-Strasse 40, D-37075 Göttingen, Germany; 2 Department of Rheumatology and Clinical Immunology, Charité University Hospital, Schumannstrasse 20/21, D-10098 Berlin, Germany; 3 Laboratory for Functional Genome Research, Charité University Hospital, Schumannstrasse 20/21, D-10098 Berlin, Germany; 4 Institute of Pathology, Moltkestrasse 32, D-54292 Trier, Germany; 5 Institute of Transfusion Medicine, Charité University Hospital, Schumannstrasse 20/21, D-10098 Berlin, Germany

## Abstract

Bone morphogenetic proteins (BMPs) have been identified as important morphogens with pleiotropic functions in regulating the development, homeostasis and repair of various tissues. The aim of this study was to characterize the expression of BMPs in synovial tissues under normal and arthritic conditions. Synovial tissue from normal donors (ND) and from patients with osteoarthritis (OA) and rheumatoid arthritis (RA) were analyzed for BMP expression by using microarray hybridization. Differential expression of BMP-4 and BMP-5 was validated by semiquantitative RT-PCR, *in situ *hybridization and immunohistochemistry. Activity of arthritis was determined by routine parameters for systemic inflammation, by histological scoring of synovitis and by semiquantitative RT-PCR of IL-1β, TNF-α, stromelysin and collagenase I in synovial tissue. Expression of BMP-4 and BMP-5 mRNA was found to be significantly decreased in synovial tissue of patients with RA in comparison with ND by microarray analysis (*p *< 0.0083 and *p *< 0.0091). Validation by PCR confirmed these data in RA (*p *< 0.002) and also revealed a significant decrease in BMP-4 and BMP-5 expression in OA compared with ND (*p *< 0.015). Furthermore, histomorphological distribution of both morphogens as determined by *in situ *hybridization and immunohistochemistry showed a dominance in the lining layer of normal tissues, whereas chronically inflamed tissue from patients with RA revealed BMP expression mainly scattered across deeper layers. In OA, these changes were less pronounced with variable distribution of BMPs in the lining and sublining layer. BMP-4 and BMP-5 are expressed in normal synovial tissue and were found decreased in OA and RA. This may suggest a role of distinct BMPs in joint homeostasis that is disturbed in inflammatory and degenerative joint diseases. In comparison with previous reports, these data underline the complex impact of these factors on homeostasis and remodeling in joint physiology and pathology.

## Introduction

In patients with rheumatoid arthritis (RA), joint pathology is mediated by typical changes in the synovial tissue. Hyperplasia of the synovial lining layer, infiltration of mononuclear cells into the sublining layer, activation of fibroblast-like synoviocytes and the production of catabolic mediators such as IL-1β, TNF-α and matrix metalloproteinases are involved in the joint destruction of patients with RA [1]. Although secondary, synovitis is also found in osteoarthritis (OA) as a response of cartilage degradation and irritation of the lining cells with cartilage matrix components. Eventually, this also induces thickening of the lining layer and aggravates the damage of articular cartilage by the release of inflammatory cytokines and destructive proteases [2].

Increases in knowledge about inflammatory cytokines and cytokine networks in chronic joint diseases has promoted the development of a new generation of biological drugs now available as inhibitors of TNF, IL-1 and others. However, little is known about mechanisms that protect and regenerate joints, although it has been shown that the progress of chronic joint diseases is decisively determined by the balance of anabolic and catabolic activities [3,4].

Bone morphogenetic proteins (BMPs) are anabolic candidates with pleiotropic functions in the development, homeostasis and repair of various tissues. Current approaches focus mainly on their ability to regenerate bone and cartilage by the induction of differentiation, apoptosis and proliferation of undifferentiated cells as well as by the stimulation of extracellular matrix formation [5,6]. These stimulatory properties led to the clinical use of recombinant BMP-7 in the treatment of bone nonunions [7]. In contrast, BMP signaling has been shown to be involved in the onset and progression of ankylosing enthesitis in spondyloarthropathies and in the induction of osteophytes in OA [8,9]. Antagonism of BMP signaling was therefore suggested as an attractive therapeutic principle [8,10].

These and other findings with opposing functional implications [5,11,12] demonstrate that the exact role of individual BMPs in degenerative joint diseases is still insufficiently understood.

In this study we focused on the expression of BMP-4 and BMP-5 in the synovial tissue of chronic joint diseases. Both proteins have a fundamental role in embryogenesis and in the induction of cartilage and bone [13,14]. Genetic and expression data suggest that BMP-5 is a key molecule in initiating the formation of particular skeletal elements in mammals [15].

In adult organisms, both BMP-4 and BMP-5, are sufficient to induce the heterotopic formation of bone and cartilage *in vivo *[16]. Moreover, diminished repair after bone fracture in BMP-5-null mutated short-ear mice suggests that BMP-5 might also be required for the growth and repair of skeletal structures after birth [15]. BMP-4 stimulates the synthesis of extracellular matrix in chondrocytes and supports the healing of bone fractures. Overexpression of BMP-4 leads to increased cartilage formation and chondrocyte differentiation without disturbing joint formation [17].

However, little is known about BMPs in synovial tissue. Lories and colleagues [18] demonstrated that BMP-2, BMP-4, BMP-6 and BMP-7 are expressed in the synovial membrane of patients with RA. BMP-2 and BMP-6, but not BMP-4 and BMP-7, are induced in fibroblast-like synoviocytes by stimulation with IL-1β and TNF-α. Moreover, intra-articular injection of BMP-2 induced fibrosis of the synovium [10], suggesting distinct effects of BMPs in synovial inflammation and joint pathology.

Here we have investigated the expression characteristics of BMP-4 and BMP-5, which were identified as differentially expressed BMPs in a comparative microarray study on synovial tissue from normal donors and patients with joint diseases. We confirmed the array data by semiquantitative PCR, *in situ *hybridization and immunohistochemistry. Decreased expression of these morphogens in the inflamed tissues and changes in their histomorphological distribution suggest that distinct members of the BMP family are involved in joint homeostasis. They may be attractive candidates for readjustment of an unbalanced intra-articular milieu dominated by destruction and lack of repair.

## Materials and methods

### Patients and tissue samples

Synovial tissue samples were obtained from patients with RA (*n *= 23) and OA (*n *= 22) undergoing open synovectomy or total joint replacement and from normal joints *post mortem *(*n *= 17) (tissue bank). Normal samples were derived from macroscopically healthy joints *post mortem*. The cause of death was cerebral bleeding or cerebral infarction. Patient characteristics and age and gender for controls are given in Table 1. No further information about the controls was made available for ethical reasons. Tissue samples for mRNA analysis by microarrays or PCR were snap-frozen in liquid nitrogen in the operating room and stored at -70°C until analyzed. Synovial tissue samples for *in situ *hybridization were embedded in OCT Tissue Tek (Miles, Elkhart, IN, USA) before being frozen. Synovial tissue samples for immunohistochemistry were embedded in paraffin. All patients with RA fulfilled the American College of Rheumatology revised criteria for definite RA [19]. The study was approved by the local ethical committee of the Charité Hospital.

### Grading of chronic synovitis

To characterize synovial disease activity and to confirm appropriate sampling before molecular analysis, the synovitis score as published by Krenn and colleagues [20,21] was applied. The histopathological inflammatory scoring system included the following three parameters: hyperplasia/enlargement of synovial lining layer (intima), activation of fibroblastic cells in the sublining stroma, and inflammatory cellular infiltration. All three parameters were graded semiquantitatively (0 = no, 1 = slight, 2 = moderate, 3 = strong) in a manner blinded to diagnosis. The values of all three parameters were added, resulting in a score between 0 and 9; 0 or 1 was interpreted as 'no synovitis', 2 or 3 as 'slight degree of synovitis', 4 to 6 as 'moderate degree of synovitis' and 7 to 9 as 'strong degree of synovitis'.

### Microarray analysis

Total RNA from synovial tissues was isolated with the Qiagen RNeasy Mini Kit in accordance with the manufacturer's protocols (Qiagen, Hilden, Germany). Total RNA was used for further microarray analysis with the oligonucleotide microarray HG-U133A (Affymetrix, Santa Clara, CA, USA) in accordance with the manufacturer's recommendations. In brief, 5 μg of total RNA was used to synthesize cDNA. Subsequently, *in vitro *transcription (ENZO Biochem, New York, NY, USA) was performed to generate biotin-labeled complementary RNA. Fragmented complementary RNA (15 μg) was hybridized to GeneChips for 16 hours at 45°C. The GeneChips were washed and stained under standardized conditions (fluidic station) and scanned on a Hewlett Packard Genearray Scanner (Affymetrix) controlled by Affymetrix MAS 5.0 software. Raw gene expression data were processed with the Affymetrix GCOS 1.2 software module in accordance with the manufacturer's default settings. Analysis was performed with Affymetrix GCOS 1.2 software to generate CEL files and the robust multiarray analysis (RMA) algorithm for signal calculation [22]. Arrays were adjusted to each other by quantile normalization in RMA.

We followed the hypothesis that BMPs might be involved in the regulation of joint homeostasis. All probe sets (*n *= 19) representing all different genes of the BMP family (*n *= 12) on the HG-U133A array were therefore selected for *t *test analysis. Adjusted *p *values for the 12 genes with Bonferroni-Holm correction (α = 0.1) were applied as the threshold of significance.

### Semiquantitative kinetic PCR

Tissues were homogenized, treated with phenol–chloroform [23] and total RNA was extracted with RNeasy spin columns (Qiagen). Single-strand cDNA was transcribed by Superscript II RT (Gibco BRL, Karlsruhe, Germany) from 5 μg of RNA in a total volume of 20 μl. The relative expression level of glyceraldehyde-3-phosphate dehydrogenase was used to normalize gene expression in each sample in different concentrations. Semiquantitative PCR was performed as described previously [1]. In brief, oligonucleotides (Gibco BRL) were selected with DNASTAR Primer Select Software (DNASTAR Inc., Madison, WI, USA). Sequences are given with GenBank accession numbers (Gibco BRL) in Table 2. All PCR reactions were performed with AmpliTaq Gold Mix (Perkin Elmer, Weiterstadt, Germany) in a reaction volume of 80 μl, amplifying at 93°C for 1 minute, 62°C for 1 minute, and 72°C for 2 minutes. For quantification of individual genes, 4 μl of each amplification reaction was removed every third cycle covering the linear detection range. Products were separated in a 1% agarose gel containing ethidium bromide and quantified densitometrically (Imager 1D&2D software; Appligene, Oncor, Illkirch, France) within the linear range comparable to the *Ct *value known from real-time PCR. The quality of amplification was controlled by the amplification efficiency as represented by the increase in product per cycle. Specificity of the PCR product was confirmed by sequencing. For graphical presentation, data are given as percentages of the glyceraldehyde-3-phosphate dehydrogenase product.

### *In situ *hybridization

*In situ *hybridization was performed as described previously [24]. BMP-4 and BMP-5 cDNA fragments were derived from the respective PCR products, cloned into pBluescript II (Stratagene, La Jolla, CA, USA) and sequenced. Digoxigenin-labeled riboprobes were transcribed with the PCR-Script Amp-Cloning Kit (Stratagene) and T3 and T7 polymerases (Roche, Mannheim, Germany). For each patient group (RA, *n *= 5; OA, *n *= 5; ND, *n *= 4), frozen sections 6 μm thick were fixed in 3% paraformaldehyde, washed in 2 × standard saline citrate (SSC) for 5 minutes, washed twice in 0.1 M triethanolamine hydrochloride, and acetylated with 0.25% acetic anhydride in 0.1 M triethanolamine hydrochloride for 30 minutes. After being washed with 1 M triethanolamine hydrochloride, sections were prehybridized for 1 hour with hybridization buffer (50% formamide, 80 μl of 50 × Denhardt's solution, 1.6 ml of 20 × SSC, 200 μl of herring sperm, 100 μl of carrier RNA) without the riboprobe. Hybridization with digoxigenin-labeled riboprobes was performed overnight in hybridization buffer at 50°C. After hybridization, sections were incubated with RNase A (40 μg/ml) for 1 hour at 37°C and subsequently washed for 15 minutes with increasing stringency (1 × SSC, 0.25 × SSC, 0.1 × SSC in 0.1% SDS) at 50°C. The staining procedure was performed with an anti-digoxigenin-alkaline-phosphatase-conjugated Fab by using 5-bromo-4-chloro-3-indolylphosphate and Nitro Blue Tetrazolium (all chemicals from Roche). Blocking was performed with 2% horse serum. Sense probes used as negative controls gave no significant signal.

### Immunohistochemical staining

BMP-4 and BMP-5 was stained in paraffin embedded tissue (RA, *n *= 4; OA, *n *= 6; ND, *n *= 4) with a modified sandwich technique as described previously [25]. Sections 4 μm thick were deparaffinized and endogenous peroxidase activity was quenched for 15 minutes with 0.3% H_2_O_2 _in methanol at room temperature. Specimens were microwave-heated for 14 minutes and incubated for 30 minutes with pooled, heat-inactivated human serum tested negative for both anti-nuclear antibodies and anti-neutrophil cytoplasmic antibodies. The primary antibodies (polyclonal goat-anti-human BMP-4 and BMP-5 antibodies; Santa Cruz Biotechnology, Santa Cruz, CA, USA) were applied for 1 hour at room temperature. Slides were incubated for 30 minutes with a horseradish-peroxidase-conjugated secondary rabbit anti-goat antibody at a dilution of 1:50, and afterwards with Dako Envision anti-rabbit antibody. Slides were incubated with the chromogenic substrate 3-amino-9-ethyl-carbazole for 5 minutes at room temperature and counterstained with hematoxylin.

### Statistical analysis

Statistical analysis was performed with GraphPad software (GraphPad Sofware Inc., San Diego, CA, USA). For microarray analysis a *t *test was used with Bonferroni-Holm correction. For comparison between RA, OA and ND (PCR), the Mann–Whitney *U *test was applied. Correlations were calculated by Spearman's rank correlation test.

## Results

### Validation of systemic and local inflammation

**Figure 1:**
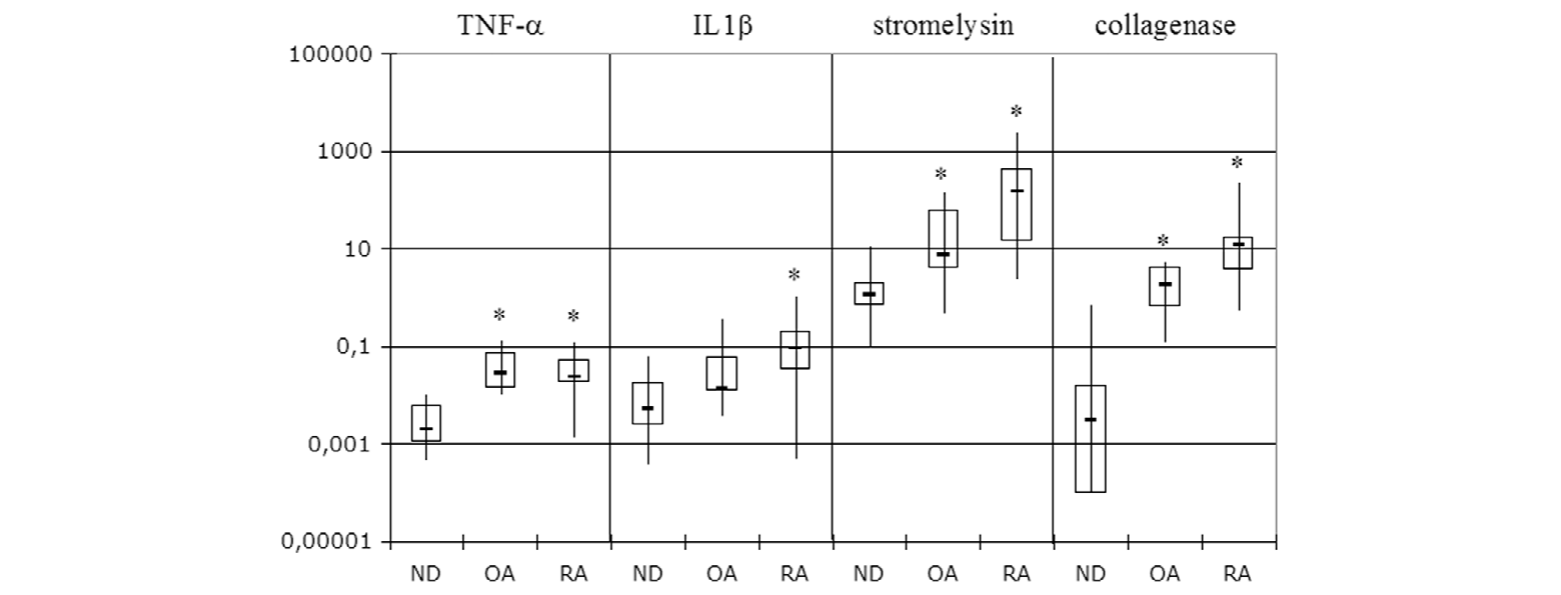
Expression of TNF-α, IL-1β, stromelysin and collagenase I in synovial tissues. Results are presented as percentage of GAPDH expression on a logarithmic scale with maximum, minimum, quartiles and median. Where indicated with an asterisk, there were significant differences from normal tissues (*p *< 0.05; Mann–Whitney). Rheumatoid arthritis (RA) versus normal donors (ND): IL-1β, *p *= 0.0097; TNF-α, *p *= 0.008; stromelysin, *p *= 0.0009; collagenase I, *p *= 0.0002. Osteoarthritis (OA) versus ND: IL-1β, *p *= 0.1451; TNF-α, *p *= 0.0013; stromelysin, *p *= 0.038; collagenase I, *p *= 0.0012. RA versus OA: IL-1β, *p *= 0.0397; TNF-α, *p *= 0.9591; stromelysin, *p *= 0.0124; collagenase I, *p *= 0.0266. GAPDH, glyceraldehyde-3-phosphate dehydrogenase.

Patients were investigated for systemic as well as local inflammation and disease activity by the analysis of blood and synovial tissue samples. Systemic inflammation was characterized by erythrocyte sedimentation rate (ESR) and C-reactive protein (CRP) (Table 1). Both markers were significantly elevated in RA in comparison with OA (CRP, *p *≤ 0.0001; ESR, *p *= 0.0001). Local inflammation and destructive activity in synovial tissue were quantified by both histological and molecular characteristics. Analysis of the tissues according to the 'synovitis score' described by Krenn and colleagues [20,21] revealed 2.1 (RA), 1.3 (OA) and 0.7 (ND) points for hyperplasia of the synovial lining layer, 1.9, 1.1 and 0.3 points for activation of the sublining stroma, and 2.1, 0.8 and 0.1 points for inflammatory infiltration in RA, OA and ND, respectively. Thus, the synovitis score – assessed in a blinded manner – was increased in all patients with RA (mean score 6.1, 'highly active synovitis') in comparison with those with ND (mean score 1.1, 'no synovitis') and patients with OA (mean score 3.2, 'mild synovitis'). For molecular characterization, expression levels of IL-1β and TNF-α as well as stromelysin and collagenase I were determined by semiquantitative PCR. These parameters were found to be highest in RA with a significantly lower expression in OA (except for TNF-α) and ND. In OA these parameters were also significantly elevated in comparison with ND except for IL-1β (Figure 1).

### Analysis of BMP-4 and BMP-5 gene expression in synovial tissue

**Figure 2:**
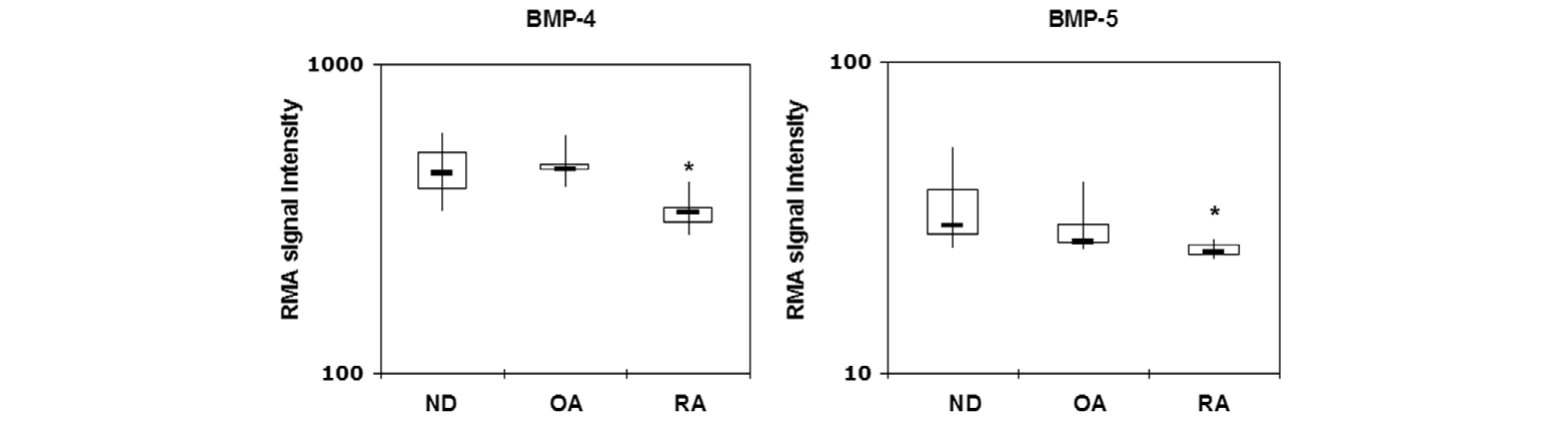
Expression of BMP-4 and BMP-5 in synovial tissues detected by microarray technique. Results are presented on a logarithmic scale with maximum, minimum, quartiles and median. Where indicated with an asterisk, there were significant differences from normal tissues (*p *< 0.05; *t *test). Rheumatoid arthritis (RA) versus normal donors (ND): bone morphogenetic protein (BMP)-4, *p *= 0.0009 (adjusted *p *≤ 0.0083); BMP-5, *p *= 0.0142 (probe set ID 205431_s_at; data not shown) and *p *= 0.006 (probe set ID 205430_at) (adjusted *p *≤ 0.009). Osteoarthritis (OA) versus ND: BMP-4, *p *= 0.854; BMP-5, *p *= 0.216 (probe set ID 205431_s_at) and *p *= 0.129 (probe set ID 205430_at) (no significance). RA versus OA: BMP-4, *p *= 0.000003 (adjusted *p *≤ 0.0083); BMP-5, *p *= 0.2391 (probe set ID 205431_s_at) and *p *= 0.026 (probe set ID 205430_at) (no significance).

Microarray analysis was performed by investigating 10 samples from each group of donors with RA, OA and normal joints. We exclusively investigated the factors of the BMP family as possible candidates involved in joint homeostasis and cartilage regeneration [5]. BMP-2 to BMP-11, BMP-14 and BMP-15 were represented on the array. In comparison with housekeeping genes, all BMPs revealed low signal levels in all samples investigated. Statistical analysis revealed significantly decreased expression of BMP-4 and BMP-5 in RA in comparison with ND. Moreover, BMP-4 was also lower in synovial tissue of patients with RA than in those with OA. There was no difference of BMP expression between OA and ND (Figure 2).

**Figure 3:**
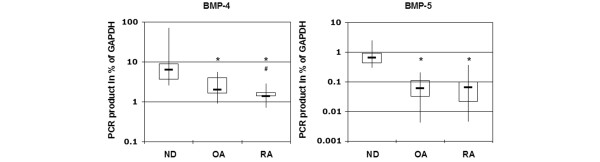
Expression of BMP-4 and BMP-5 in synovial tissues detected by semiquantitative PCR. Results are presented as percentage of GAPDH expression on a logarithmic scale with maximum, minimum, quartiles and median. Where indicated, there were significant differences from normal tissues (asterisk) or osteoarthritis (OA) (hash sign) (*p *< 0.05, Mann–Whitney). Rheumatoid arthritis (RA) versus normal donors (ND): bone morphogenetic protein (BMP)-4, *p *= 0.0005; BMP-5, *p *= 0.0016. OA versus ND: BMP-4, *p *= 0.0143; BMP-5, *p *= 0.0011. RA versus OA: BMP-4, *p *= 0.0180; BMP-5, *p *= 0.9215. GAPDH, glyceraldehyde-3-phosphate dehydrogenase.

This differential expression of BMP-4 and BMP-5 as determined by microarray technique was verified by semiquantitative PCR (Figure 3). A significantly reduced expression of both BMPs was found in OA and RA tissue in comparison with normal synovial tissue (*p *< 0.015). Expression of BMP-4 in RA synovial tissue was also lower than in tissues from patients with OA (*p *< 0.02). For BMP-4, there was no overlap between the ranges of RA and ND expression values: all values of RA tissues were lower than the minimum level found in ND tissues. In OA, expression values of 5 of 12 synovial tissues were within the range of ND expression values. For BMP-5, expression in all patient samples except those from one RA donor were below the range of expression in ND tissues. Thus, PCR analysis confirmed the results for RA versus ND as determined by microarray hybridization.

Correlation analysis of BMP-4 and BMP-5 with each other and with markers of inflammation was performed by combining the data from RA and OA donor groups for the respective parameters. BMP-4 was found to decrease with rising systemic inflammation as represented by ESR (*r *= -0.4184, *p *= 0.0298) and C-reactive protein (*r *= -0.5808, *p *= 0.0012) as well as with disease duration (*r *= -0.6343, *p *= 0.0005). Furthermore, expression of BMP-5 was negatively correlated with an increase in TNF-α expression (*r *= -0.4739, *p *= 0.0167).

### *In situ *hybridization and immunohistochemistry

**Figure 4:**
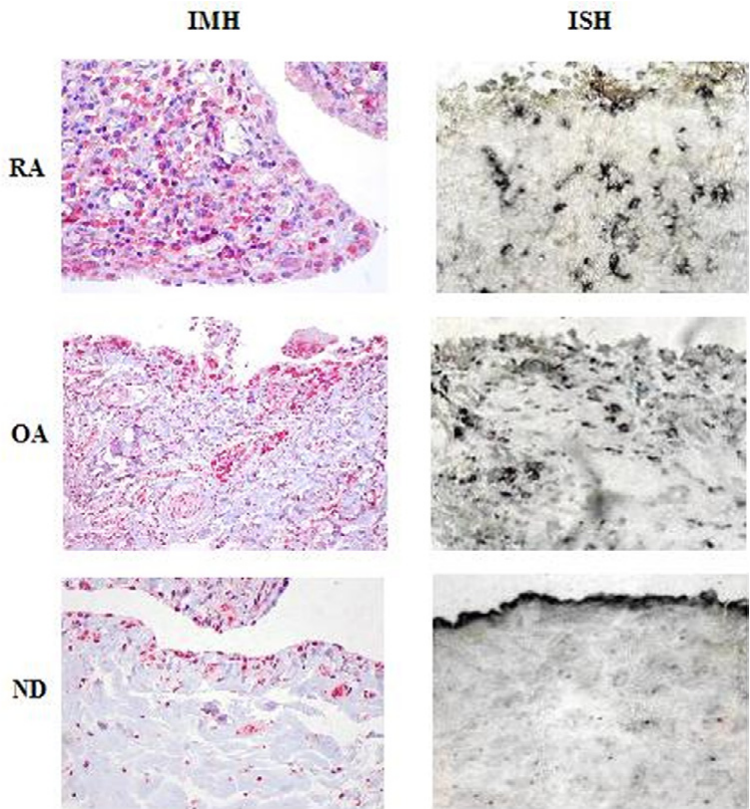
Immunohistochemistry and *in situ *hybridization of synovial tissues for BMP-4. In normal synovial tissue the expression of bone morphogenetic protein (BMP) is localized to the synovial lining layer. In rheumatoid arthritis (RA) and osteoarthritis (OA) tissue samples BMP-4 is expressed less by cells of the superficial synovial layer but more by cells scattered in deeper layers. Original magnifications: immunohistochemistry (IMH): RA, normal donors (ND) ×40, OA ×20; *in situ *hybridization (ISH): RA, OA, ND ×40.

**Figure 5:**
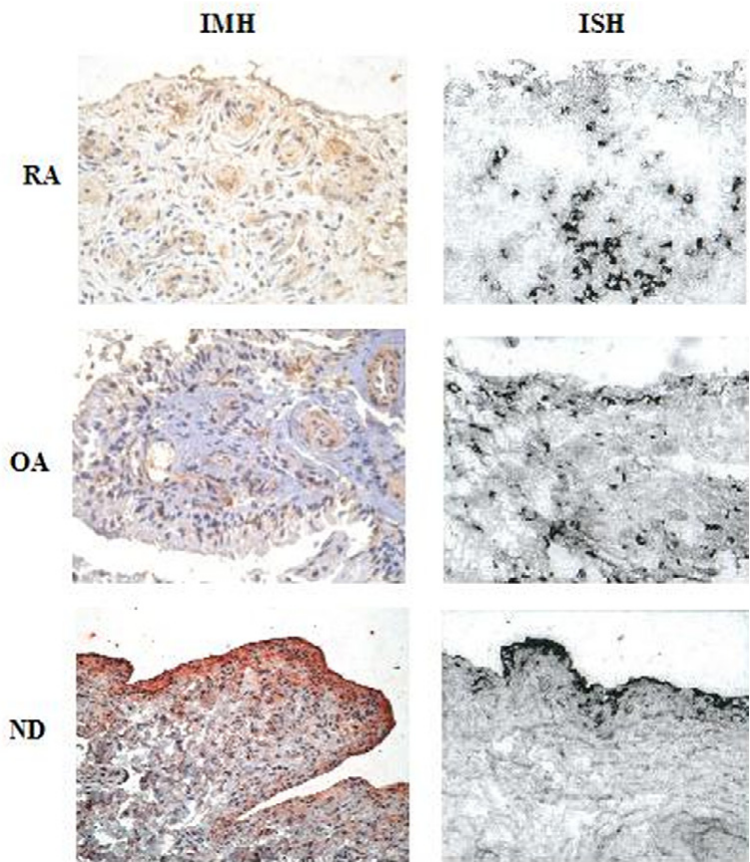
Immunohistochemistry (IMH) and *in situ *hybridization (ISH) of synovial tissues for BMP-5. Histomorphological distribution of BMP-5 is comparable to that of BMP-4 (Figure 4). Original magnifications: immunohistochemistry (IMH): rheumatoid arthritis (RA), osteoarthritis (OA), normal donors (ND) ×20; *in situ *hybridization (ISH): RA, OA, ND ×40. BMP, bone morphogenetic protein.

Synovial tissue of patients with RA, OA and ND was analyzed to localize the morphological site of BMP-4 and BMP-5 expression by mRNA *in situ *hybridization and immunohistochemistry (Figures 4 and 5). Both techniques present only qualitative morphological results and do not reflect the quantity of transcripts.

**Figure 6:**
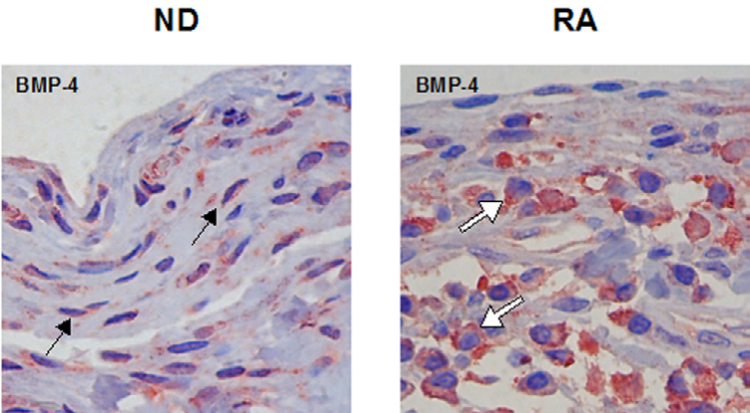
Expression of BMP-4 in fibroblastoid (black arrow) and macrophagocytic (white arrow) cells by immunohistochemistry. Original magnifications: normal donors (ND), rheumatoid arthritis (RA) ×100. BMP, bone morphogenetic protein.

**Figure 7:**
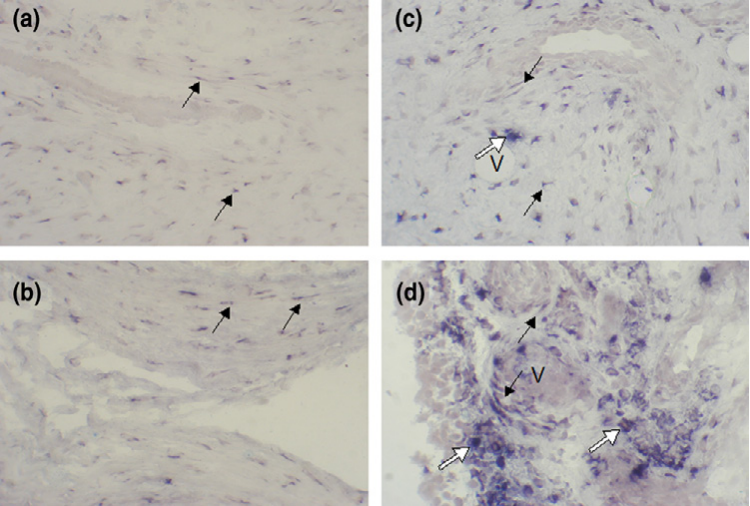
Fibroblasts (black arrows) expressing bone morphogenetic protein (BMP)-4 **(a) **and BMP-5 **(b) **in areas with fibrosis in osteoarthritis synovial tissue (original magnification ×20). Macrophagocytic (white arrows) and fibroblastoid (black arrows) appearance of cells adjacent to vessels (V) expressing BMP-4 **(c) **and BMP-5 **(d) **in rheumatoid arthritis synovial tissues (original magnification ×40).

*In situ *hybridization in normal synovial tissue (ND) revealed BMP-4 and BMP-5 expression predominantly on the surface of the synovial membrane. However, in RA and OA tissues BMP-4 and BMP-5 were less dominant in the superficial layer but were also found in cells of the sublining layer. Both morphogens were mostly localized to cells with large nuclei or spindle-like shape (Figure 6). Especially in OA samples with areas of fibrous tissue formation, cells were positively stained for morphogen transcripts (Figure 7a,b). Perivascular cell infiltrates also contained positive cells with large nuclei along with positive cells of spindle-like appearance, thus resembling macrophage and fibroblastoid morphology, respectively (Figure 7c,d).

To confirm the results of *in situ *hybridization, antibody staining for BMP-4 and BMP-5 protein was performed in independent samples. Synovial tissues of all three groups (RA, OA and ND) revealed positive results. The sites of expression of both morphogens were identical to those found by *in situ *hybridization. Both methods therefore documented independently that BMP-4 and BMP-5 expression is related to the synovial lining layer in ND and more to the sublining layer in RA and OA patients (Figures 4 and 5).

## Discussion

Inflammation and destruction are leading pathomechanisms in chronic joint diseases. In recent years, however, aspects of regeneration and homeostasis have become more and more important. Members of the TGF-β family, especially BMPs, are pivotal factors in skeletal tissue development and may contribute to the repair of various other tissues. We investigated the expression of BMPs in the synovial tissue compartment under normal and pathologic conditions by using microarray technology. All BMPs from BMP-2 to BMP-11, BMP-14 and BMP-15 revealed low to very low signal levels. Of these experiments, BMP-4 and BMP-5 were significantly decreased in RA in comparison with ND. This difference was confirmed by semiquantitative PCR. In addition, PCR analysis revealed a reduced expression of BMP-4 and BMP-5 in OA tissue in comparison with normal tissue. This variance of BMP expression levels in OA tissue in comparison with normal or RA synovial tissue may be explained by technical differences in sensitivity and resolution between PCR and microarray hybridization. However, the groups analyzed by PCR and microarray were independent. BMP expression in OA may therefore be more variable than that in RA. Immunostaining in normal donors revealed the expression of both BMPs predominantly in the synovial lining layer, whereas in patients with RA the expression was more frequently found in the sublining layer. A decrease in BMP-4 and BMP-5 in RA and OA could be correlated with markers of systemic and in part with markers of local inflammation as well as with disease duration. A relation of BMP suppression to therapy with steroids and disease-modifying anti-rheumatic drugs administered only in RA was excluded because BMP expression in synovium of OA patients was affected similarly, although to a lesser extent.

Expression of BMPs in synovial tissues was investigated recently by Lories and colleagues [18]. They compared synovium from RA and spondyloarthropathies with synovium from traumatic joint diseases and found BMP-2 and BMP-6 to be expressed most consistently with a calculated relative expression in the range 0.002 to 0.2% compared with β-actin. This confirms our own observations of a low expression level in the synovial tissue compartment. Similarly to their results, we could not detect differential expression of BMP-2 and BMP-6 mRNA in RA compared with normal tissue. *In vitro*, however, Lories and colleagues found an increase in BMP-2 and BMP-6 expression on stimulation of cultivated synovial fibroblasts with TNF-α and IL-1β. These data seem in part controversial to our observation that in synovial tissue the expression of the BMPs investigated (BMP-4 and BMP-5) was decreased. In addition, BMPs were negatively correlated with local or systemic parameters of inflammation as well as the duration of the disease. This discrepancy might depend on differences in the biological function and regulation of individual members of the BMP family. In fact, Lories and colleagues [18] also reported that BMP-4, in contrast to BMP-2 and BMP-6, was not increased by stimulation with IL-1β or TNF-α. Furthermore, local differences between stimulatory and inhibitory mechanisms for BMP production could explain our observed differences in the histomorphological distribution of BMP-expressing cells in RA compared with controls. A similar distribution and predominant expression of different BMPs in fibroblastoid and macrophagocytic cells was also shown by Lories and colleagues [18] and van Lent and colleagues [26].

That BMPs might provide a beneficial effect on joint repair can be assumed from their role in joint development [27], their induction of chondrogenic differentiation in adult mesenchymal stem cells [28,29] and their effect on cartilage formation in tissue engineering with chondrocytes [5]. Similarly, the decrease in BMP-7 expression and the increase in BMP antagonists found in osteoarthritic cartilage suggests that a loss of BMP signal might reduce the regenerative capacity of cartilage [12,30]. However, the role of BMPs in the homeostasis of joints and the regeneration of cartilage is still unclear. BMP-2 was found to be increased in osteoarthritic cartilage and stimulated in culture with the proinflammatory cytokines IL-1 and TNF [31]. In contrast, other BMPs were unchanged [32]. Furthermore, the expression of BMP-6 and BMP-7 was also decreased in articular cartilage of TNF-transgenic mice, suggesting that loss of BMP expression could be also involved in chronic inflammatory and not only degenerative joint diseases [33]. The overall decrease in BMP-4 and BMP-5 in the synovial membrane therefore presents a new and additional aspect in the imbalance of joint homeostasis in chronic joint diseases.

As well as a possibly beneficial effect of BMPs on arthritic joints, intra-articular TGF-β injection was shown to induce osteophyte formation, a typical morphological change in OA [34]. Moreover, recent studies suggested that other factors such as BMP-2 and BMP-4 might be involved as downstream mediators of the TGF-β effect and that these BMPs might be released by macrophages of the synovial lining layer [26]. However, these data are derived from a mouse model with TGF-β injected into normal joints. Furthermore, the dosage of TGF-β applied was at least 1,000-fold higher than the TGF-β concentration found in normal or even osteoarthritic joint synovia [35]. Nevertheless, these data demonstrate that uncontrolled high levels of morphogens may exert a negative influence. It is intriguing that inhibition of BMP signalling in a papain-induced OA mouse model could prevent osteophyte formation and synovial fibrosis but at the same time increased the loss of proteoglycan from the cartilage matrix, thereby certainly promoting the damage of the joint surface [10].

Thus, regenerative triggers in the treatment of joint diseases will depend on a balanced action of stimulators and inhibitors of BMP signalling with precise modulation of specific BMPs. The histomorphological distribution may be also important. Expression in deeper layers as seen in the samples of our RA and OA patients may influence predominantly cells of the surrounding tissue, thereby contributing to synovial fibrosis. In contrast, expression in the synovial lining layer may be more relevant for stable or increased levels of BMP in the synovial fluid, where these morphogens may potentially influence articular cartilage. As BMP-4 and BMP-5 were found to be decreased in the synovium and their expression was attributed to the synovial lining layer in normal joints, they could be favorable candidates for therapeutic application. Nevertheless, it will be important to understand precisely the network of morphogen action and regulation in the joint, because injection of BMP-2 induced osteophyte formation in a murine model [9]. Thus, the interaction of BMPs and inhibitors not only in the synovium but also in cartilage has to be elucidated. Although studies in developmental biology have contributed considerably to the understanding of the BMP network [27], the role of these morphogens in adult tissues is still unclear.

## Conclusion

BMP-4 and BMP-5 are expressed in normal synovial tissue and were found to be decreased in OA and RA. Furthermore, the histomorphological distribution of both morphogens showed a dominance in the lining layer in the normal tissue, whereas their expression in RA and OA tissue was also scattered across deeper layers. These results suggest that BMP-4 and BMP-5 may be important in joint homeostasis and are therefore potential candidates for joint regeneration.

## Abbreviations

BMP = bone morphogenetic protein; CRP = C-reactive protein; ESR = erythrocyte sedimentation rate; IL = interleukin; ND = normal donors; OA = osteoarthritis; PCR = polymerase chain reaction; RA = rheumatoid arthritis; RT = reverse transcriptase; SSC = standard saline citrate; TNF = tumor necrosis factor.

## Competing interests

The authors declare that they have no competing interests.

## Authors' contributions

CPB and TH performed patient recruitment, PCR, immunohistochemistry and data interpretation and drafted the manuscript. UU was involved in *in situ *hybridization and PCR. VK was involved in patient recruitment and performed the 'synovitis score'. AP and CK conducted part of the patient recruitment and data evaluation. FS, GAM and GRB provided substantial input into data evaluation. All authors read and approved the final manuscript.

## Figures and Tables

**Table 1:** Clinical characteristics of patients

	Microarray			PCR		
	RA (*n *= 10)	OA (*n *= 10)	ND (*n *= 10)	RA (*n *= 13)	OA (*n *= 12)	ND (*n *= 7)
Median age (range), years	60 (39–73)	67 (58–78)	57 (40–76)	69 (29–74)	67 (53–83)	51 (34–61)
Male/female	2/8	1/9	7/3	5/8	2/10	4/3
Median disease duration (range), years	7 (2–37)	10 (1–19)	NA	10 (4–30)	5 (1–38)	NA
Median ESR (range), mm/h	33 (12–78)	20 (10–60)	NA	32 (22–86)	11 (2–29)	NA
Median CRP (range), mg/l	22.1 (6.1–113.3)	6.4 (2–19)	NA	19 (5.9–50.8)	4.8 (0–9.3)	NA
Rheumatoid factor positive, *n *(range, units)	4 (47–400)	1 (29)	-	10 (15–2,450)	-	-
Patients receiving steroids, *n*	8	0	NA	9	0	0
All patients receiving DMARDs, *n*	9	0	NA	9	0	0
Patients receiving MTX	7	0	NA	6	0	0
Patients receiving NSAIDs, *n*	6	5	NA	8	4	0
Synovial tissue from knee	5	10	10	6	5	7
Hip	0	0	0	3	7	0
Hand synovectomy	3	0	0	3	0	0
Elbow synovectomy	2	0	0	1	0	0

CRP, C-reactive protein; DMARDs, disease-modifying anti-rheumatic drugs; ESR, erythrocyte sedimentation rate; NA, not applicable; ND, normal donors; OA, osteoarthritis; RA, rheumatoid arthritis.

**Table 2:** Oligonucleotides

mRNA	GenBank accession number	Oligonucleotide (5'→3') (up/down)	Product size (bp)	Annealing temperature (°C)
GAPDH	M33197	ATG GGG AAG GTG AAG GTC GGA GTC GAC GCC TGC TTC ACC ACC TTC TTG	797	62
TNF-α	M10988	CTC TGG CCC AGG CAG TCA GA GGC GTT TGG GAA GGT TGG AT	519	62
IL-1β	M15330	CAC CTG TAC GAT CAC TGA ACT GCAC GGC TGG GGA TTG GCC TGC AA	674	60
MMP-1	X05231	CTG CTG CTG TTC TGG GGT GTG GTG GTG GGC CGA TGG GCT GGA CAG	793	62
MMP-3	J03209	TGG AGC TGC AAG GGG TGA GGA CAC CAG GCG GAA CCG AGT CAG GTC TGT	691	62
BMP-4	M22490	ACC CGG GAG AAG CAG CCA AAC TAT AGC GGC ACC CAC ATC CCT CTA CTA	553	62
BMP-5	M60314	GGC ATC CTT GGC AGA AGA GAC CA ACT GCG TCC ATC CCC TGT TTC TG	535	62

BMP, bone morphogenetic protein; GAPDH, glyceraldehyde-3-phosphate dehydrogenase; MMP, matrix metalloproteinase; MMP-1, collagenase I; MMP-3, stromelysin.
